# School Hygiene

**Published:** 1893-04-15

**Authors:** 


*For Hospitals, Asylums, Infirmaries, Dispensaries, Medical Practitioners, Students, Nurses, and the Charitable Public.*

Vol. XIV.—No. 342.] April 15, 1893.

### School Hygiene.

There are certain things that the public does with a prompt will and spontaneous enthusiasm; there are other things which it will not do at all, except under the persuasion of whip and spur. Scientists tell us with the nicest exactitude what ought to be done, and they often explain to us the evil consequences which may result from neglect of their advice. But what they fail to do is to furnish compelling motives. They do not seem to understand that one of the weakest of all motive powers is a remote penalty, which is not even an absolute certainty, but only a doubtful possibility. They might, one would think, take a lesson from the history of theological dogma. In the days of Dante the whole population of Europe believed with unquestioning faith in the reality of an Inferno, of which the poet's presentation was but a shadow and a feeble dream. Yet at no period in the world's history did vice of all kinds more universally prevail. The penalty was too remote and uncertain to act as a checking motive on the passions of the time. Dr. Caldwell Smith has presented the public with an admirable pamphlet on School Hygiene. But he has entirely failed to supply the motive power which shall convert his excellent principles into concrete realities.

From the age of four to the age of thirteen the children of the great mass of people who live in this country spend the larger part of their day within the precincts and in the atmosphere of the public elementary school. School is the soil in which children grow, and the climate in which they live. Soil and climate: these constitute the two prime conditions of vegetable life, and their analogues are equally fundamental for animal and human life. Suitable soil and a fine climate produce the rarest, most vigorous, and most beautiful forms of vegetation which we can see. In worthless soils and severe climates vegetation almost entirely ceases. The parallel holds exactly for children, and indeed for animal life generally. What we have to do is to persuade and convince all classes of Englishmen and Englishwomen that the school to which our children are so confidingly sent is sometimes a place of growth and life, and sometimes—perhaps frequently—a place of withering and death.

Dr. Caldwell Smith informs us, and nobody doubts it, that the site on which a school is built, or to be built, is of prime importance. But how many of the public elementary schools which have been erected within even the last twenty-five years, are standing on sites which will conserve child health and promote child development? Nay, how many of the schools which are frequented by the children of the wealthy will satisfy these modest and extremely elementary requirements? More, in detail we are told that a low site is dangerous, whilst a high site is wholesome. The reasons why a low site is dangerous—namely, ground water and ground air in shifting excess—are so obvious that one would think every grown-up person would be familiar with them, whilst school managers should have such an instinctive dislike for them that no proprietor of land need offer such sites for sale for school purposes. That is the instructed condition of mind in which the public ought now to be found if the elementary lessons of hygienic science had taken the smallest root in the general intelligence.

With equal detail and clearness, Dr. Caldwell Smith expounds the other obvious and reasonable principles which go to make up the science of school hygiene. He insists, for example, upon the subordination of every other desideratum to the one idea of abundant air and light. He tells us that consumption has markedly increased among the children of the poor since the passing of Mr. Forster's Act, and he has little doubt that crowded and insanitary schools are responsible for the discreditable fact. In some German schools so little as 42 cubic feet of space is the allowance for each child, whereas even in casual wards the Local Government Board insists upon 300 cubic feet for each sleeper, and a properly-constructed hospital secures 1,800 cubic feet for every patient. How important is the arrangement of windows in a school may be judged by the fact that shortsightedness among children prevails to an alarming extent, and seems to be in inverse proportion to the abundance and proper incidence of the supply of light. For example, Kohn found in Germany that among the children of village schools, where light was abundant, only 1·4 per cent. were myopic, whilst in the corresponding class in large towns as many as 10 per cent., and in some cases even 20 per cent., were afflicted with short sight. Among the better classes an even worse condition of things prevails. Conrad examined 3,036 eyes, and found that while among the lower class 11·1 only were myopic, among the more educated 62·1 were in that condition. Of 410 students of the University of Breslau, 60 per cent. were short-sighted. Is it possible for hygienic scientists, with these facts before them, to insist too strongly upon the amplest abundance of uninterrupted light for all classes of children and young people?

We do not often associate spinal curvature with school life. But it is obvious that we should. It is stated by Gulenberg that of all the cases of spinal disease other than those of a constitutional origin no fewer than 90 per cent. originate at school. This is a disgrace at once to our humanity and our common sense. Young children should sit upon seats with backs, in order that they may have that support which the growing and flexible spine absolutely needs. Our grandfathers' idea that seats with backs made children lazy is too ridiculous to be seriously protested against. And yet it is not improbable that half the population among the less educated classes hold firmly by that idea to this day.

On the subject of "hours of study" for children, Professor Caldwell Smith speaks with great decision, and with his protest we express, as we have often expressed before, the most entire agreement. He says: "The total time devoted to study should be from two and a-half to three hours for children under seven years of age; three or three and a-half hours below ten years; and below twelve, four hours should be the maximum. In the younger classes the use of the Ling or Swedish system should be compulsory. The teacher should, every 15 minutes, ring a bell, and the children should then perform a few muscular movements in regular succession to the accompaniment of brisk and cheerful music. Regarding home lessons, I do not think they should be given at all to the younger children."

Now no man or woman of sense will deny the reasonableness of a single one of Dr. Caldwell Smith's contentions. But notwithstanding this, myopia, spinal curvature, tuberculosis, and other preventable diseases prevail to a most unhappy extent among all classes of children in this country, and still more in Germany. We are committing crimes against our children every day so long as these things are allowed to continue. But it is the children, not ourselves, which must bear the punishment. What does a man owe to his children? What does a nation owe? These are large questions; and questions which go to the roots of family life and a worthy morality. The commandments of science, we insist, are as obligatory upon us as the commandments of religion. To the cultured and reasoning mind they are the commandments of religion. What obligations do we know that surpass in importance or in personal imperativeness those we owe to our children? None! The man who fails of his highest and most intelligent duty to his children vitiates thereby every other good work of his life. Dr. Caldwell Smith, and others like him, have supplied us with excellent practical science for the happiness and health of those who are dependent upon us. Will they now devote themselves to the creation of a motive power which shall make their science universally prevail?

